# Letter to Editor “Dual-energy CT for the detection of skull base invasion in nasopharyngeal carcinoma: comparison of simulated single-energy CT and MRI”

**DOI:** 10.1186/s13244-023-01510-w

**Published:** 2023-12-17

**Authors:** Takashi Hiyama, Hirofumi Kuno

**Affiliations:** Department of Diagnostic Radiology, National Cancer Center Hospital East, 6-5-1, Kashiwanoha, Kashiwa, Chiba 277-8577 Japan

**Keywords:** Dual‑energy CT, Nasopharyngeal carcinoma, Skull base

Dear Editor in Chief,

We recently read the article by Dr. Yang Zhan et al., titled “Dual-energy CT for the detection of skull base invasion in nasopharyngeal carcinoma: comparison of simulated single-energy CT and MRI” published in Insight into Imaging with great interest [1]. The authors have addressed a clinically crucial topic in a scientific manner, and we appreciate their efforts. However, we have a few concerns that we would like the authors to clarify.

Our first concern pertains to the iodine concentration in sclerotic lesions. In the material decomposition method, since tissue is assumed to be composed of two or three substances (fat, soft tissue, iodine, etc.), iodine and bone may not be entirely distinguishable. For example, assuming that the substance is composed of iodine and water, the bone might appear in both the iodine and the water image, leading to the misinterpretation that the bone is composed of these substances [2, 3]. Consequently, we are concerned that the iodine concentration in the skull base may not accurately reflect the actual amount of iodine. Regarding Figure 4, the iodine concentration in the bone cortex of the right base of the pterygoid process and the bony wall of the bilateral maxillary sinuses may be due to the inability to distinguish between bone and iodine. The sclerotic lesion on the left pterygoid process in this case does not show contrast enhancement on the MRI, but positive finding on the iodine overlay images is clearly seen, possibly due to bone sclerosis. Additionally, in Figure 2, the iodine concentration is higher in the sclerotic invasion than in the control group, whereas the iodine concentration is lower in the osteolytic invasion. Theoretically, iodine distribution should be higher in lytic bone than in normal bone, reflecting iodine enhancement to the tumor. Is it possible that this is because the bone component of normal bone is misidentified as iodine, resulting in higher iodine concentration in sclerotic lesions and lower iodine concentration in osteolytic lesions?

Our second concern relates to the standard reference. Since the iodine concentrations on DECT and MRI findings, which are being evaluated in this study, have been used as the criteria for the standard reference, we are concerned that they may influence the results. As mentioned above, the iodine image in the bone may not accurately reflect the actual amount of iodine. Furthermore, the lesion was considered positive for invasion if skull base invasions persisted or became larger in images after 6 months in this study. Persistence implies no change, so why were the lesions that did not change considered to be tumor invasion? The fact that there is no change with treatment or course of the disease raises the possibility that imaging findings are not due to tumor invasion.

Lastly, the definition of a score of 4 (probably positive) in the imaging evaluation section is not provided.

Yours sincerely.

## Data Availability

Not applicable.
